# Reduced physical activity during submarine deployment: health and performance consequences and feasible countermeasures—a narrative review

**DOI:** 10.1093/joccuh/uiag026

**Published:** 2026-05-18

**Authors:** Benjamin J C Kirk, Georgios Mavropalias, Anthony J Blazevich, Jodie L Cochrane Wilkie, Aus Molan, Kazunori Nosaka

**Affiliations:** School of Medical and Health Sciences, Edith Cowan University, Joondalup, WA, Australia; School of Health Sciences, The University of Notre Dame Australia, Sydney, NSW, Australia; School of Medical and Health Sciences, Edith Cowan University, Joondalup, WA, Australia; School of Medical and Health Sciences, Edith Cowan University, Joondalup, WA, Australia; School of Medical and Health Sciences, Edith Cowan University, Joondalup, WA, Australia; Physical Activity, Sport and Exercise Research Theme, Faculty of Health, Southern Cross University, Gold Coast, QLD, Australia; Exercise Medicine Research Institute, Edith Cowan University, Joondalup, WA, Australia; PathWest Laboratory Medicine, Perth, WA, Australia; School of Medical and Health Sciences, Edith Cowan University, Joondalup, WA, Australia

**Keywords:** submariners, physical inactivity, submarine deployment, occupational health, minimal-dose exercise, eccentric exercise

## Abstract

**Objectives:**

Submarine environments pose unique challenges to maintaining physical activity due to space constraints, operational demands, and prolonged confinement. This review aims to synthesize existing literature on the health and performance consequences of reduced physical activity in submarine personnel and to examine feasible strategies to mitigate these effects during deployment.

**Methods:**

A narrative overview methodology was adopted, with peer-reviewed studies identified from PubMed, Scopus, and Google Scholar. Articles examining physical activity patterns, health outcomes, and exercise or movement-based interventions in submarine environments and comparable confined operational settings were included.

**Results:**

Submarine deployments are consistently associated with reduced physical activity, low step counts, and limited opportunities for structured exercise. Prolonged inactivity during deployment may contribute to unfavorable changes in body composition, including increases in fat mass, potential reductions in muscle mass, and in some contexts, alterations in bone health. Collectively, these changes may increase longer-term cardiometabolic and musculoskeletal risk, particularly with repeated deployments. Practical, low-burden strategies such as increasing daily movement, no-load or bodyweight resistance exercise, and minimal-dose eccentric-focused training appear feasible within submarine constraints and may help preserve health and physical function.

**Conclusions:**

Maintaining physical health during submarine deployments requires proactive, context-specific approaches compatible with the operational environment. Low-resource exercise strategies that emphasize maintenance of activity, rather than performance optimization, may provide a practical means of mitigating health and performance declines during deployment. Further research is needed to determine the long-term effectiveness and implementation of these approaches in submarine populations.

## Teaser

**Key points**
 

- **What is already known on this topic:** Submarine service involves prolonged exposure to confined, isolated environments that substantially restrict physical activity. While reduced physical activity during deployment has been reported, the broader health and performance consequences of prolonged inactivity in submarine personnel have not been comprehensively synthesized.
- **What this review adds:** This narrative review synthesizes available evidence indicating that submarine deployments are associated with marked reductions in physical activity and a range of physiological changes that may collectively increase cardiometabolic and musculoskeletal health risk over time. These include unfavorable alterations in body composition, potential reductions in muscle mass, and, in some contexts, altered bone health. The review also identifies low-burden, evidence-informed approaches that appear compatible with the operational constraints of submarine environments, including increasing daily movement and bodyweight or no-load resistance exercise.
- **How this review might affect research, practice, or policy:** These findings highlight the need to prioritize maintenance-focused physical activity strategies that are compatible with operational demands during submarine deployments. Emphasizing low-resource, scalable exercise approaches may help guide future research, inform health promotion practices, and support occupational decision-making aimed at preserving the long-term health and readiness of submarine personnel.

## 1. Introduction

Exercise plays a fundamental role in maintaining health, physical fitness, and functional capacity across occupational settings.^1^^,^^2^ Physical fitness, encompassing strength, endurance, mobility, and flexibility, is a key determinant of work capability and is associated with a reduced risk of chronic disease.^3^ Higher levels of fitness are also linked to a lower incidence of musculoskeletal injury,^4^^,^^5^ contributing to fewer sick days and reduced health care costs and productivity-related costs.^6^ Additionally, physical fitness contributes to greater resilience, a critical attribute in military personnel.^7^

Public health guidelines recommend that adults accumulate 150 to 300 minutes of moderate-intensity or 75 to 150 minutes of vigorous-intensity physical activity each week (or an equivalent combination of both moderate and vigorous activities), incorporate muscle-strengthening activities on at least 2 days per week, and minimize prolonged sitting.^2^ However, fewer than 20% of adults meet these recommendations.^8^ This highlights the challenge of sustaining adequate physical activity even under normal living conditions, and underscores the heightened difficulty faced in extreme or highly constrained occupational environments.

Submarine service represents one such environment. Due to the covert nature of operations, submarines are typically smaller than other military vessels, resulting in prolonged periods of working and living in confined and isolated spaces.^9^ These spatial constraints substantially limit opportunities for structured physical activity. Recent survey data from Royal Australian Navy submariners indicate that submarine deployment is associated with a profound reduction in physical activity, with total activity decreasing by more than 80% compared with land-based conditions, and a majority of personnel reporting little or no structured exercise while at sea. ^10^ In addition to space constraints, submariners face several environmental and operational factors that can adversely impact health and well-being, including elevated CO_2_ concentrations,^11^ absence of natural light, artificial atmospheres, limited hygiene opportunities due to restricted water supply, rotating shift schedules (commonly 6 hours on-duty followed by 6 hours off-duty) across deployments that may span weeks to several months, and centrally provisioned diets that rely heavily on packaged and shelf-stable foods, with limited availability of fresh items.^12^^,^^13^ Collectively, these factors may discourage regular exercise participation during deployment, contributing to reductions in physical activity and increased sedentary behavior.

The aim of this narrative review is to synthesize existing literature on the health and performance consequences of reduced physical activity during submarine deployments and to explore practical, context-specific strategies for sustaining physical health and well-being in this unique occupational environment.

## 2. Methods

Given the limited scope and heterogeneity of the literature specific to submariner populations, a formal systematic review was not considered appropriate. Instead, a narrative overview methodology was adopted using the typological framework described by Grant and Booth.^14^ This approach was selected because the breadth of the topic, the limited volume of submarine-specific literature, and the likelihood that relevant studies were not consistently labeled under a single search framework at the time of publication preclude the application of a single structured search strategy. The primary aim was to collate and synthesize studies examining physical activity patterns, health outcomes, and exercise or movement-based interventions in submarine and related occupational contexts.

Searches were conducted across PubMed, Scopus, and Google Scholar to ensure broad coverage of the relevant literature published up to February 2026. Given the breadth of this narrative review and the limited volume of literature specific to submarine populations, searches were conducted iteratively across topic areas rather than through a single structured search string. Example search strings included: (“submarine” OR “submariner”) AND (“physical activity” OR “exercise” OR “fitness”); (“submarine” OR “submariner”) AND (“body composition” OR “muscle” OR “bone” OR “cardiometabolic”); and (“eccentric exercise” OR “minimal dose training” OR “bodyweight exercise”) AND (“confined environment” OR “military” OR “occupational”). A snowballing strategy was additionally employed, whereby reference lists of identified articles were reviewed to locate relevant sources not captured by database searches.

Inclusion criteria prioritized studies conducted within submarine environments or involving submariner participants. Where data were limited, studies from comparable settings (eg, naval deployments, shipboard environments, or other isolated and confined occupational settings) were considered. General population studies were included only when required to contextualize physiological mechanisms or intervention feasibility. As this was a narrative review, no formal risk of bias or study quality assessments were undertaken. Of the 109 references cited in this manuscript, 19 (17%) are derived directly from submarine environments or involve submariner participants; 23 (21%) are drawn from comparable occupational settings; and 67 (61%) are general population studies included to contextualize physiological mechanisms or the feasibility of proposed interventions. A summary of the 19 studies conducted within submarine environments or involving submariner participants is provided in Table 1.

**Table 1:** Summary of studies conducted within submarine environments or involving submariner participants included in this review.

Author (year)	Study design	Population/submarine type	Sample	Deployment duration	Outcomes measured	Key findings
**Kirk et al (2025)**	Cross-sectional survey	Royal Australian Navy; Collins class diesel-electric submarine	*n* = 21 (18 male, 3 female)	Varied (land vs sea conditions)	Physical activity patterns; exercise participation; sedentary behavior; sleep quality; perceived barriers	Physical activity decreased >80% during deployment vs land (745 to 118 min/wk); 62% reported no structured exercise at sea; sleep quality declined 37%; sitting time increased 51% on workdays; top barriers: water restrictions (57%), limited space/equipment (43%), fatigue (38%)
**Watenpaugh et al (2004)**	Technical report	US Navy; nuclear-powered attack submarines (SSN)	Not reported (survey of 10 fitness coordinators)	Not specified	Exercise options and rationale aboard attack submarines	35% ± 12% of submariners exercised regularly while underway; severe space constraints identified as primary barrier; additional barriers included time constraints, fatigue, noise restrictions, and limited equipment; proposed new exercise options for attack submarines
**Schillaci (1965)**	Technical report	US Navy submarine personnel (general)	Not reported	Not specified	Chemical constituents of submarine atmosphere; CO_2_ concentrations; atmospheric control systems	Documented elevated CO_2_ in submarine atmosphere; outlined atmospheric control systems including oxygen sources, CO_2_ removal methods (LiOH, MEA scrubber), and contaminant monitoring equipment
**Gregg & Jankosky (2012)**	Cross-sectional	Male US Navy personnel; mixed vessel types (smaller submarines, larger submarines, aircraft carriers)	*n* = 26 341 males (5439 on smaller submarines; 3707 on larger submarines; 17 195 on aircraft carriers)	Not specified	Physical readiness test failure rates; BMI; overweight and obesity prevalence	Overweight/obesity prevalence higher on smaller submarines (69%) vs larger submarines (66%) vs aircraft carriers (63%); obese personnel had 14 times higher odds of failing physical readiness test compared with normal weight personnel
**Rietjens et al (2020)**	Longitudinal observational	Royal Netherlands Navy; diesel-electric (~68 m)	*n* = 13 males (body composition data: *n* = 10)	3 mo	Energy expenditure (DLW); body composition (deuterium dilution); physical activity level (PAL)	Net positive energy balance +221 kcal/d; PAL 1.54 ± 0.21 (*n* = 10); fat-free mass significantly decreased (−4.1 ± 3.3 kg, *P* = .003); fat mass increased nonsignificantly (+1.8 kg, *P* = .06); vitamin D and B12 concentrations decreased significantly
**Bhutani et al (2015)**	Longitudinal observational	Indian Navy submariners; vessel type not specified	*n* = 42 males (officers and sailors)	26 d	Body composition (bioimpedance): body weight, fat %, muscle mass %	Body weight increased significantly (+0.5%, 70.71 to 71.06 kg, *P* < .05); fat % increased 2.6% (*P* < .05); muscle mass % decreased 0.7% (*P* < .05); BMI increased 0.5% (*P* = .058)
**Gasier et al (2016)**	Longitudinal observational	US Navy; nuclear-powered ballistic missile submarine (SSBN; USS Nevada Gold Crew, SSBN733)	*n* = 53 males (aged 20-39 y)	~92 d (3-mo patrol)	Cardiometabolic biomarkers (lipids, glucose, insulin, adipokines, inflammatory chemokines); body composition (air displacement plethysmography); dietary and physical activity assessment	Pre-patrol: 62% obese (BF% ≥25%), 30% of obese group met metabolic syndrome criteria. Post-patrol: body mass (−5%) and fat mass (−11%) reduced in obese group due to reduced energy intake; fat-free mass maintained in both groups; modest improvements in serum lipids; inflammatory markers IP-10 and MCP-1 decreased; 43% remained obese post-patrol; 18% still met metabolic syndrome criteria
**Gunner et al (2020)**	Longitudinal observational	Royal Navy; nuclear-powered submarine	*n* = 153 (122 submerged, 31 nonsubmerged; 150 male, 3 female)	6 or 12 wk (submerged crews); 12 wk (shore control)	Serum lipids (total cholesterol, TG, LDL-C, HDL-C, non-HDL-C); glucose; insulin; anthropometrics; self-reported dietary intake and activity	Submerged crews: significant decreases in body weight (−1.4 ± 4.2 kg), total cholesterol, triglycerides, glucose, and insulin post-patrol. Shore control: body weight increased (+1.9 ± 1.8 kg); total cholesterol increased. Modest favorable cardiometabolic changes in submerged crews attributed primarily to negative energy balance and weight loss
**Kang & Song (2017)**	Cross-sectional	Korean Navy; submariners vs nonsubmarine naval personnel	*n* = 590 males (410 submariners, 180 nonsubmariners)	Career-based (≥6 mo submarine service to qualify as submariner)	Multimorbidity prevalence (≥2 chronic conditions); disease burden (Cumulative Illness Rating Scale, CIRS); diagnosed health conditions from ICD medical records	Multimorbidity prevalence 32.2% in submariners vs 11.7% in nonsubmariners; submariners had significantly higher CIRS scores across all age groups; submarine service ≥1 year associated with higher multimorbidity after adjusting for age, alcohol, smoking, and rank; no dose–response relationship with duration of submarine service
**Maguire et al (2023)**	Retrospective cohort	US Navy submariners; Virginia, Los Angeles, Seawolf, and Ohio class submarines	*n* = 26 014 (25 977 male, 37 female)	Final year of active duty service (2009-2018)	ICD diagnosis codes during final year of active duty; prevalence rates per 1000 submariners across 17 ICD categories	Largest categories: nervous system (20%) and musculoskeletal (17%). Highest operationally relevant prevalence rates per 1000 submariners: joint disorders (180), sleep disorders (134), back disorders (128), lipid disorders (95), essential hypertension (76); three mental health conditions among top 20 diagnoses
**Hughes et al (2024)**	Retrospective cohort	US Navy submariners; mixed submarine classes (SSN, SSBN, SSGN)	1283 confirmed MEDEVACs (2012-2020); 974 linked to personnel records (965 male, 7 female)	January 2012 to December 2020 (9-y observation period)	MEDEVAC causes classified by 3 major diagnostic categories (psychiatric, injury, medical/noninjury); demographic and occupational characteristics	Annual average: 143 MEDEVACs. Causes: medical/noninjury 57.3% (GI conditions 12.2%, renal/GU 8.7%, dental 6.7%); psychiatric 24.3% (suicidal ideation 11.8%); injury 18.4% (blunt trauma 9.0%). Younger submariners (21-25 y) overrepresented in psychiatric MEDEVACs (52.4% vs 37.5% medical); older submariners (≥35 y) overrepresented in medical cases (17.0% vs 2.7% psychiatric)
**Holy et al (2012)**	Longitudinal observational	French Navy; nuclear-powered ballistic missile submarine (SSBN)	*n* = 40 males (winter patrol *n* = 20; summer patrol *n* = 20)	60 d	Bone metabolism markers (BAP, ICTP); vitamin D status (25(OH)D, 1,25(OH)_2_D); acid–base balance (pH, PCO_2_, HCO_3_^−^); mineral homeostasis (Ca^2+^, Pi, PTH)	Both groups developed mild chronic respiratory acidosis-like episode with impaired bone remodeling coupling (Days 1-41). Winter patrol submariners (vitamin D deficient pre-patrol, 25(OH)D ~17 ng/mL) showed no recovery of bone remodeling coupling by patrol end; summer patrol submariners (vitamin D sufficient, ~36 ng/mL) showed partial recovery. Pre-boarding vitamin D supplementation recommended for winter patrols
**Davies & Morris (1979)**	Narrative review	Nuclear submariners (general)	Not reported	Not specified	CO_2_ and vitamin D effects on calcium metabolism	Review of calcium excretion data showing rapid fall to ~50% of pre-patrol levels during long patrols, attributed partly to CO_2_ but predominantly to vitamin D deficiency from sunlight absence; hypovitaminosis D effects predominate late in patrol and post-return; implications for bone health discussed
**Luria et al (2010)**	Longitudinal observational	Israeli Navy; Dolphin class diesel-electric submarine	*n* = 32 young males (mean age 22.8 ± 3.8 y)	30 d	Bone strength (QUS tibial SOS); bone turnover markers (BAP, PINP, TRAP5b, CTx); endocrine regulators (Ca^2+^, PTH, 25(OH)D)	Tibial SOS decreased significantly post-patrol (4081 → 4044 m/s, ~0.9%; *P* < .02); continued declining to 4011 m/s at 4 wk post-return (~1.7% below baseline; *P* < .0005); baseline SOS regained at 6-mo follow-up. Overall bone turnover reduced; serum 25(OH)D decreased ~15% (*P* < .0005)
**Gasier et al (2014a)**	Randomized double-blind placebo-controlled trial	US Navy; nuclear-powered ballistic missile submarine (SSBN; USS Nevada Gold Crew)	*n* = 53 males (placebo *n* = 16; 1000 IU/d *n* = 20; 2000 IU/d *n* = 17)	92 d (departed fall, returned winter)	Vitamin D status (25(OH)D); bone turnover markers (BAP, CTx, osteocalcin); tibial bone structure and strength (pQCT at 4% and 66% sites); body composition (air displacement plethysmography)	49% were vitamin D insufficient (<50 nmol/L) pre-patrol. 25(OH)D increased in all groups post-patrol including placebo, driven largely by weight/fat loss rather than supplementation alone (no significant group × time interaction, *P* = 0.06). pQCT revealed small but significant increases in tibial vBMD (+0.6%) and bone strength index, independent of supplementation. Short-term skeletal health not negatively affected by 3 mo of submergence
**Gasier et al (2014b)**	Cross-sectional	Current and former US submariners; mixed submarine types (diesel, SSN, SSBN)	*n* = 462 males (aged 20-91 y)	Career-based (cumulative sea time)	Bone mineral content and density at lumbar spine and proximal femur (DXA)	No association between cumulative sea time and BMC or BMD overall; serving on a diesel submarine was independently associated with lower BMD at total hip and femoral neck; prevalence of osteoporosis in submariners ≥50 y not significantly different from general population norms
**Helmhout & Rietjens (2017)**	Longitudinal observational	Royal Netherlands Navy; diesel-electric submarine	Training phase: *n* = 19; operational deployment: sleep group *n* = 14, nutrition group *n* = 16	10-wk training phase; 14-wk operational deployment	Physical fitness (12-min run); body composition (deuterium dilution); energy expenditure (DLW and activity tracker); sleep quantity/quality; mental performance (PVT); stress indicators (cortisol/melatonin); blood and urine markers (lipids, vitamins, minerals)	Training phase: no significant changes in fitness or body composition. Operational deployment: body fat % significantly increased (21.9% ± 3.2% → 27.0% ± 6.1%); fat-free mass significantly decreased (65.6 ± 8.3 → 61.6 ± 6.1 kg); triglycerides and free fatty acids significantly decreased; large interindividual differences in sleep and cognitive performance in both phases
**Choi et al (2010)**	Observational (pedometry)	Republic of Korea Navy; 209-class diesel-electric submarine	*n* = 109 males (submarine crew *n* = 76 from two 209-class submarines; submarine command *n* = 33)	~1 mo (pedometer worn across deployed and stationed periods)	Daily step counts; ambulatory activity patterns	Ambulatory activity declined markedly during deployment: ~2000 steps/d deployed (2211 ± 121 steps/d overall) vs 8181 ± 269 steps/d when stationed (*P* < .01). Deployed activity falls within sedentary category (<5000 steps/d). Age, rank, and BMI did not significantly affect ambulatory activity
**Horn et al (2003)**	Longitudinal observational (prospective serial survey)	US Navy; nuclear-powered attack submarine (SSN)	*n* = 122 males	101 d (continuously submerged)	Self-reported health complaints; hygiene habits; exercise frequency and type; body weight; self-medication patterns	68% exercised ≥3 times/wk during deployment (vs 65% on shore); exercising group lost mean 3.9 lb vs nonexercising group gained 0.8 lb (ns); most prevalent complaints: stuffy nose (41%), trouble sleeping (38%), backache (31%); hygiene declined (showering 8.6 → 5.3 times/wk); mean atmospheric CO_2_ 0.49%

Abbreviations: BAP, bone-specific alkaline phosphatase; BF%, body fat percentage; BMC, bone mineral content; BMD, bone mineral density; BMI, body mass index; CIRS, Cumulative Illness Rating Scale; CTx, C-terminal telopeptide; DLW, doubly labeled water; DXA, dual-energy x-ray absorptiometry; GI, gastrointestinal; GU, genitourinary; HDL-C, high-density lipoprotein cholesterol; ICD, International Classification of Diseases; ICTP, cross-linked C-telopeptide of type I collagen; IP-10, interferon γ-induced protein 10; LDL-C, low-density lipoprotein cholesterol; MEA, monoethanolamine; MEDEVAC, medical evacuation; ns, not significant; MCP-1, monocyte chemoattractant protein-1; PAL, physical activity level; PCO_2_, partial pressure of carbon dioxide; Pi, inorganic phosphate; PINP, procollagen type I N-terminal propeptide; pQCT, peripheral quantitative computed tomography; PTH, parathyroid hormone; PVT, Psychomotor Vigilance Test; QUS, quantitative ultrasound; SOS, speed of sound; SSBN, nuclear-powered ballistic missile submarine; SSGN, nuclear-powered guided missile submarine; SSN, nuclear-powered attack submarine; TG, triglycerides; TRAP5b, tartrate-resistant acid phosphatase 5b; vBMD, volumetric bone mineral density.

## 3. Results

### 3.1 Body composition

Overweight and obesity, defined as excessive fat accumulation that impairs health, are key risk factors for cardiometabolic disease, musculoskeletal disorders, and reduced physical performance.^3^^,^^15^ These conditions arise primarily from chronic energy imbalance, whereby caloric intake exceeds energy expenditure over time.^15^ In occupational settings characterized by limited opportunities for physical activity, such imbalances may be exacerbated.

Military organizations, including the Australian Defence Force (ADF), commonly use body mass index (BMI) as a screening tool to assess body composition.^16^ For ADF entry, a BMI range of 18.5-30 kg/m^2^ is preferred, with an upper allowable limit of 32.9 kg/m^2^.^17^ However, BMI has recognized limitations, particularly in individuals with greater lean mass, and supplementary measures such as waist circumference may provide a more accurate assessment of health risk.^17^

Consistent with trends in the general population, the prevalence of overweight and obesity has increased among military personnel.^18^ In the Australian Army, approximately 23.3% of personnel are classified as overweight and 4.5% as obese.^19^ Excess body mass in military populations has been associated with increased risk of injury and illness, higher health care utilization, reduced physical fitness performance (eg, slower run times, lower upper-body and core strength), and greater likelihood of medical discharge.^12^^,^^20–22^

Submariners may be at particular risk of unfavorable body composition changes due to the combined effects of constrained physical activity and deployment-specific lifestyle factors. A study of US Navy personnel reported moderate but statistically significantly higher rates of overweight and obesity among crews serving on smaller submarines (69%) compared with larger submarines (66%) and aircraft carriers (63%),^23^ suggesting a potential link between vessel size, physical activity opportunities, and body composition. Beyond health-related concerns, excess body mass in submarine settings may have disproportionate operational implications. Reduced mobility associated with excess weight may impair the ability to perform physically demanding tasks, to navigate confined compartments, or to respond efficiently during emergency procedures.^24^ In isolated environments with limited access to medical support, elevated cardiometabolic risk associated with excess adiposity may further compound the potential consequences of excess body mass during deployment.

Empirical data indicate that submarine deployments are associated with positive energy balance and gradual fat mass gain. Rietjens et al^25^ reported a net positive energy balance of 221 ± 506 kcal/d and a mean physical activity level (PAL) of 1.54 ± 0.21 in 10 male submariners during a 3-month deployment. This PAL is lower than values typically reported for the general population (~1.7)^26^ and was associated with an estimated increase in fat mass of approximately 2.5%, equivalent to ~1.8 kg, over the deployment period. When averaged across the deployment duration, this equates to an approximate fat mass gain of ~0.1 to 0.2 kg/wk and is consistent with changes reported during other submarine deployments.^27^

It remains unclear whether submariners return to baseline body composition following deployment. A study of US Navy personnel found that body mass increased post-deployment and continued to rise with successive deployments.^28^ These findings suggest that submariners, like the broader Navy population, are unlikely to return to pre-deployment body mass levels between missions. Instead, body mass may increase progressively across a submariner’s career,^29^ consistent with age-related trends observed in the general population.^30^ This trajectory increases the likelihood of failing physical fitness assessments, sustaining injuries, and being discharged from service.^22^^,^^31^^,^^32^

Because changes in body mass are fundamentally driven by the balance between energy intake and expenditure,^33^ deployment-related weight gain may theoretically be mitigated through dietary modification, increased physical activity, or a combination of both.^2^ However, the spatial and logistical constraints of submarine environments limit the feasibility of traditional exercise approaches, highlighting the need for context-appropriate strategies to manage energy balance during deployment.

### 3.2 Cardiometabolic health and blood biomarkers

In addition to changes in body composition, several studies have examined cardiometabolic health indicators in submariners. Acute deployment studies generally report mixed or modest changes in individual blood biomarkers, such as lipid profiles or glucose regulation, with many parameters remaining within clinical reference ranges.^34^^,^^35^ However, these short-term findings should be interpreted within a broader occupational context. Larger cross-sectional and retrospective cohort studies indicate that submariners exhibit a high prevalence of cardiometabolic risk factors over the course of their careers, including disorders of lipid metabolism, overweight and obesity, and essential hypertension.^36^^,^^37^ Notably, endocrine and circulatory conditions feature prominently among diagnoses associated with separation from submarine service and medical evacuation, suggesting that the cumulative effects of prolonged occupational exposure, such as repeated periods of physical inactivity and constrained movement, may contribute meaningfully to long-term health outcomes.^37^^,^^38^ Collectively, these findings highlight a distinction between short-term physiological responses during deployment and longer-term disease risk that may emerge across repeated patrols and extended service.

### 3.3 Bone health

Bone health is strongly influenced by mechanical loading and physical activity. Bone mineral density (BMD), which accounts for approximately 70% of bone strength, is commonly used to assess skeletal health and diagnose conditions such as osteoporosis.^39^ Bone density typically peaks during the second and third decades of life and declines progressively with age in the absence of sufficient mechanical loading, such as resistance or weight-bearing exercise.^40^ The amount of bone mass attained and maintained during adulthood is therefore a key determinant of skeletal health later in life, with prolonged exposure to unfavorable conditions increasing the risk of osteoporotic fracture in older age.^41^

Submariners face several environmental and occupational factors that can negatively impact bone mass. Limited exposure to sunlight may impair vitamin D metabolism, which plays a critical role in bone formation.^42^^,^^43^ Prolonged exposure to elevated carbon dioxide (CO_2_) levels in the recirculated submarine atmosphere can also lead to respiratory acidosis, a reduction in body fluid pH caused by increased CO_2_ partial pressure, which has been shown to reduce bone formation and increase bone resorption.^44–46^ In addition, space limitations and noise control requirements may restrict exercise options during deployment, reducing the feasibility of resistance or impact-based training modalities known to support skeletal health.^47^

One study specifically examined submariner bone health using quantitative ultrasound to assess tibial speed of sound (SOS) before and after 30 days of submersion on a diesel-electric submarine.^48^ The study reported a 0.91% reduction in bone quality (from 4081 ± 17 to 4044 ± 16 m/s, *P* < .02) following the 30-day deployment, equivalent to a decline of approximately 0.21% per week. Bone quality continued to decline by a further 1.7% 4 weeks after returning to shore (4011 ± 1 m/s, *P* < .0005), which the authors attributed to delayed bone remodeling or a lag in the resumption of habitual physical activity post-deployment. For context, a separate study in healthy American Caucasian men aged 20 to 90 years reported no significant decline in tibial SOS between the ages of 20 and 80.^49^ Relative to these age-related norms, the magnitude and persistence of the reductions observed by Luria et al^48^ suggest deployment-related changes in bone quality that exceed those expected from aging alone and therefore warrant further investigation. Additionally, as recovery of bone parameters was not observed until approximately 6 months post-deployment, repeated submersions within this timeframe may pose cumulative risks to skeletal health.

In contrast to these findings, evidence from a longer 92-day deployment aboard a submerged ballistic missile (SSBN) submarine reported a small but statistically significant increase in BMD (0.6 ± 2.1%, *P* < .05), assessed using peripheral quantitative computed tomography (pQCT).^50^ The authors suggested that this increase may reflect early-stage exercise adaptation, supported by concurrent reductions in both whole body mass and fat mass alongside fat-free mass maintenance. Collectively, these changes are consistent with the possibility that participants maintained or increased habitual physical activity during the patrol. However, the absence of objective physical activity measurements limits interpretation and prevents determination of the exercise dose required to maintain or enhance BMD during submarine deployment.

To examine the long-term effects of submarine service on bone health, Gasier et al^51^ conducted a cross-sectional study involving 462 current and former US submariners aged 20 to 91 years. Bone mineral content (BMC) and BMD were assessed using dual-energy X-ray absorptiometry (DXA), a gold standard method for skeletal assessment.^52^ The study found no association between cumulative time spent at sea and either BMC or BMD, nor evidence of an increased risk of low BMD or osteoporosis later in life among submariners. One exception was observed in individuals who had served on diesel-powered submarines (as examined by Luria et al^48^), who exhibited lower BMD at the total hip and femoral neck. Compared with modern nuclear-powered submarines, including SSBN platforms, diesel submarines typically operate with less stringent atmospheric control, and higher onboard CO_2_ concentrations have been reported (approximately 0.8%-1.2% vs 0.6%-1.0%).^11^^,^^44^^,^^48^ Elevated CO_2_ exposure has been associated with reduced bone formation and increased resorption,^44^^,^^46^^,^^53^ which may partially explain the site-specific reductions in BMD observed in this subgroup. However, despite these differences, no increased prevalence of osteopenia or osteoporosis was identified, suggesting limited clinical significance at the population level.

Overall, the unique environmental factors encountered during submarine deployments have the potential to compromise bone health. However, available evidence suggests that any short-term deterioration in bone integrity during deployment may be reversible during in-port or shore-based duties, as physical activity levels and mechanical loading return toward habitual levels. Nevertheless, implementing strategies to support physical activity and skeletal loading during patrols may be beneficial for preserving bone health across a submariner’s career.

### 3.4 Muscle mass

Skeletal muscle plays a central role in force production, movement, and metabolic health, with greater muscle mass associated with higher strength and improved metabolic function.^54^^,^^55^ Maintenance of muscle mass is therefore critical for physical performance and long-term health.

Skeletal muscle is highly plastic and responds readily to changes in mechanical loading. Muscle size increases with training and declines with inactivity or unloading.^56^ In adulthood, muscle mass is generally maintained when regular mechanical stimulus is preserved, but declines progressively with aging and reductions in physical activity.^57^^,^^58^ Consequently, environments that restrict daily movement or resistance-based activity may place individuals at risk of muscle mass loss.

Several studies have reported reductions in lean body mass during submarine patrols,^25^^,^^27^^,^^48^^,^^59^ whereas others have observed no significant changes.^34^^,^^50^ These mixed findings may reflect differences in physical activity levels during patrol, baseline body composition of participants, nutritional intake, and sleep quality. For example, participants in the study by Gasier et al^34^ were stationed aboard a large (~170 m) US nuclear-powered (SSBN) submarine whereas those in Rietjens et al^25^ served on a smaller (~68 m) diesel-electric submarine. Larger vessels may afford greater space and opportunity for incidental or structured physical activity compared with the more confined environment of diesel-electric submarines. Additionally, a substantial proportion of participants in the study by Gasier et al^34^ (62%) were classified as obese, which may have increased daily mechanical loading of the locomotor muscles due to higher body mass. This increased loading could provide a training-like stimulus that contributes to the preservation of muscle mass during deployment.^60^

Where muscle loss occurs during submarine patrols, it is unlikely to result in immediate impairment of occupational performance. However, reductions in muscle mass may contribute to slower recovery from illness, delayed wound healing, and reductions in resting metabolic rate, which could cumulatively compromise operational capacity.^61^ Over the longer term, chronic reductions in muscle mass, such as those observed in sarcopenia, have been associated with adverse endocrine and inflammatory profiles, increased risk of physical disability, diminished quality of life, and higher health care utilization.^55^^,^^61^

Given the plasticity of the musculoskeletal system, regular mechanical loading is essential for the preservation of muscle tissue.^58^ Resistance training represents one of the most effective countermeasures against muscle loss associated with inactivity and, in some contexts, has demonstrated greater efficacy than pharmacological interventions.^62^ However, no studies have specifically examined resistance training interventions in submariners before, during, or after deployment. Consequently, little is known about the type, frequency, or intensity of training that may be feasible in this environment, or the minimum effective dose required to attenuate muscle loss during patrols, highlighting an important area for future research.

### 3.5 Muscle strength

Muscular strength is defined as the ability to exert force against an external resistance.^63^ Higher strength levels are associated with improved performance of occupational tasks, including physically demanding activities such as casualty evacuation and load carriage as well as precision-based tasks such as weapon handling and shooting.^64–66^ In addition, greater strength is associated with a lower risk of musculoskeletal injury, resulting in fewer sick days and reduced health care costs and compensation-related costs.^4–6^

Muscle strength is influenced by both neural and structural factors. Neural contributors include the capacity to voluntarily activate muscle and coordinate motor unit recruitment,^67^^,^^68^ while structural and mechanical factors include muscle cross-sectional area, architecture, and musculotendinous stiffness.^69–71^ Both components are sensitive to habitual use and mechanical loading.^56^

To our knowledge, no studies have directly examined changes in muscular strength before, during, or after submarine deployment. Evidence from detraining studies indicates that maximal strength (eg, one-repetition maximum) can generally be maintained for several weeks following cessation of resistance training in both younger and older adults.^72–74^ However, more pronounced declines in strength have been observed following longer periods of inactivity exceeding approximately 12 weeks, particularly in older individuals.^75^ Although direct evidence in submariners is lacking, similar patterns of strength loss may occur during prolonged deployments, especially given the low physical activity levels typically reported during patrols.^9^^,^^76^ The magnitude of strength loss is likely to vary according to factors such as baseline fitness, age, and the type and frequency of physical activity performed while at sea.

### 3.6 Detraining effects on strength and muscle mass

Regarding strength retention during detraining, the literature reports mixed findings. Some studies describe a near-complete loss of training-induced strength gains after 6, 8, or 12 months without training,^77^^,^^78^ whereas others report partial retention of strength following 3 to 5 months^79^ or even up to 12 months of detraining.^80^ These discrepancies likely reflect differences in participant characteristics (eg, age, training status, body composition) and training program variables (eg, duration, intensity, and exercise modality).^77^ Age appears to be a particularly influential factor, as older individuals tend to experience more rapid declines in strength during periods of detraining.^75^ Nevertheless, across studies, strength losses generally occur more slowly than the gains achieved during resistance training.

In contrast, the effects of detraining on muscle mass appear to be more transient than those on strength. Most studies indicate that muscle volume and cross-sectional area (CSA) return to pre-training levels within approximately 24 to 31 weeks following cessation of resistance exercise.^81–85^ This pattern has been reported in both young and older adults,^83^^,^^84^ although young men (20-30 years) may demonstrate slightly greater resilience to muscle loss than young women and older individuals. For example, Melnyk et al^84^ observed a 4% reduction in mid-thigh CSA in young men after 31 weeks of detraining, compared with declines of 7%, 6%, and 12% in young women, older men, and older women, respectively.

However, the extent of muscle mass loss during detraining is not uniform across studies, and some evidence suggests that muscle mass may be partially preserved for extended periods, particularly following higher training volumes. For example, Psilander et al^86^ reported minimal sex-related differences in CSA loss after 20 weeks of detraining, indicating that age and prior training history may be more influential determinants than sex alone. More broadly, several studies indicate that the rate of muscle loss during detraining is slower than the rate of muscle gain achieved during training, suggesting a degree of muscular resilience. Supporting this, Staron et al^87^ demonstrated that type IIab + IIb fiber CSA in young women increased by 46.5% following 20 weeks of resistance training, yet declined by only 14.2% after a subsequent 30-32-week detraining period. Notably, both Psilander et al^86^ and Staron et al^87^ employed higher training volumes than studies reporting more rapid muscle loss (eg, Ivey et al^83^ and Melnyk et al^84^), which may help to explain the greater retention observed and account for discrepancies in the literature.

Most of the aforementioned detraining studies have been conducted in previously untrained participants. In well-trained individuals, longer periods of detraining (eg, ≥20 weeks) may be associated with a more rapid initial decline in muscle volume, largely attributable to reductions in muscle glycogen and other noncontractile substrates.^88^ However, structural adaptations such as myofibrillar hypertrophy are likely to persist above sedentary levels, even following extended periods without training.

## 4. Strategies to support health and fitness onboard submarines

### 4.1 Pre-deployment

Various exercise modalities are known to support the maintenance of health and fitness in the general population. Resistance training and high-impact activities such as running and jumping help preserve muscle and bone mass, while moderate-intensity aerobic exercise is effective for supporting cardiovascular fitness. In general populations, these exercise modalities are commonly recommended at frequencies of approximately 2 to 3 sessions per week as part of public health guidelines aimed at maintaining health and physical fitness.^89^ However, exercise onboard submarines is particularly challenging due to confined physical space, limited equipment availability, time constraints, restricted water use, lack of instruction or exercise education, and the need to minimize noise and vibration for tactical reasons.

Given these constraints, one potential strategy is to periodize an intensive block of physical training in the weeks or months leading up to deployment. This approach would involve maximizing physical capacity prior to patrol, with the expectation that some detraining may occur while at sea. Previous research suggests that both muscle and bone mass are relatively resilient to short-term periods of inactivity. For instance, lower-body strength in young resistance-trained men was unchanged after 2 weeks of detraining.^73^ In another study, participants who completed three 6-week training blocks interspersed with two 3-week detraining periods achieved similar hypertrophy and strength gains to those who trained continuously for 24 weeks, suggesting that up to 3 weeks of detraining may not negatively affect strength or hypertrophic outcomes.^72^ However, the feasibility of this strategy is limited in practice. In the lead-up to deployment, work-related duties often intensify, with sailors frequently working 12-hour shifts, leaving little time or desire for structured physical training. Additionally, this approach may not be effective for longer patrols. Deployments in the submarine literature typically range from approximately 1 to 3 months (Table 1), and extended inactivity may lead to meaningful losses in muscle mass and strength, particularly at the longer end of this range, though the precise rate and magnitude of maladaptation over time remain uncertain.^9^

### 4.2 During deployment

**Figure 1:**
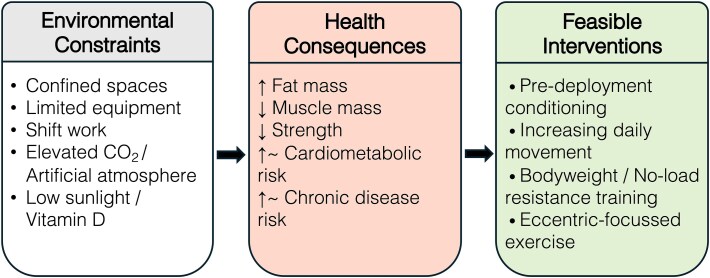
Overview of key environmental constraints encountered during submarine deployment and their associated health consequences, including adverse changes in body composition, musculoskeletal function, and chronic disease risk. Suggested intervention strategies are shown on the right and represent practical, low-resource approaches that may help mitigate these effects while accommodating the unique limitations of the submarine environment. Note: ~ indicates a possible or variable effect based on mixed findings in the literature.

Maintaining physical fitness during submarine deployments is inherently difficult due to restricted space, limited access to equipment, and demanding shift schedules.^9^ As a result, traditional training programs are often impractical, and opportunities for spontaneous movement are minimal. Accordingly, strategies that require minimal space, equipment, and time commitment are needed to mitigate declines in health and performance while at sea. While the approaches described below draw on evidence from comparable and general population settings rather than submarine-specific research, this section explores feasible options, including increasing daily movement, bodyweight and no-load resistance training, and minimal-dose eccentric exercise, that may support musculoskeletal and cardiovascular health onboard submarines. Figure 1 provides a conceptual overview of how the submarine environment contributes to health and performance risks during deployment and highlights feasible countermeasures that may help mitigate these effects. Importantly, in the context of submarine deployment, preserving existing physical capacity is likely to be a more appropriate objective than optimizing performance; therefore, exercise strategies that deliver a minimal yet sufficient mechanical stimulus are likely to be most suitable.

#### 4.2.1 Increasing daily movement

One simple strategy to mitigate physical deconditioning is to increase general movement throughout the day. Step count provides a practical proxy for physical activity, with 7500 to 10 000 steps per day associated with health benefits.^90^ However, during a diesel-electric submarine patrol, crew members were reported to accumulate just 2000 to 3000 steps per day,^76^ highlighting the highly sedentary nature of the environment. In contrast, Horn et al^91^ reported that exercise frequency appeared largely maintained during a 101-day deployment aboard a nuclear-powered attack submarine, with 68% of crew exercising 3 or more times per week, a finding that may reflect the comparatively greater space available on larger nuclear vessels, as discussed elsewhere in this review. Increasing daily step count, where operationally feasible, may help preserve metabolic health and physical conditioning.^92^ Nevertheless, space constraints and operational duties often limit opportunities for walking, necessitating alternative strategies to maintain physical activity during deployment.

#### 4.2.2 Bodyweight and no-load resistance exercises

When equipment is unavailable, bodyweight and no-load resistance exercises offer practical alternatives for maintaining musculoskeletal function. Bodyweight training, in which one’s own mass is used as resistance, can help maintain strength and functional capacity^93^ and may be progressed by manipulating leverage, repetitions, tempo, or through the use of simple load implements such as weighted vests.^94^^,^^95^ “No-load” resistance training, where maximal voluntary contractions are performed through a full range of motion without external load, has also been shown to induce hypertrophic adaptations comparable to traditional resistance training. For example, Counts et al^96^ reported similar muscle hypertrophy following 6 weeks of no-load biceps curls compared with loaded training at 70% one-repetition maximum in previously untrained participants. Together, these findings suggest that bodyweight and no-load resistance exercises might be feasible for maintaining musculoskeletal function in submarine environments where space, equipment, and training time are limited.

#### 4.2.3 Minimal-dose eccentric exercise

Eccentric exercise involves lengthening muscle actions performed under load, such as lowering the body during a squat or descending stairs. Compared with concentric actions, eccentric contractions are characterized by a lower metabolic cost for a given force output, and the capacity to generate high forces when required.^97–100^ A growing body of evidence supports the use of low-volume eccentric training to induce strength and health benefits with minimal time and physical strain.^101^ This makes eccentric exercise particularly attractive in operational settings where space, exertion tolerance, and recovery resources are limited.

Recent studies in sedentary individuals have demonstrated that a brief, 5-minute daily routine of slow eccentric-biased bodyweight exercise performed at home can improve muscle strength, strength endurance, flexibility, and selected health indicators over periods of 4 to 8 weeks, with high adherence and minimal perceived strain.^102^^,^^103^ These programs consisted of simple movements such as chair squats and wall push-ups performed with prolonged eccentric (lowering) phases of approximately 5 seconds and comparatively brief concentric return phases. The protocols required no equipment, were perceived as tolerable, and achieved high adherence, with benefits sustained at 12-month follow-up. Collectively, these findings suggest that minimal-dose eccentric-biased exercise may offer a feasible and scalable approach to maintaining physical health during submarine deployment.

In older adults, eccentric-dominant exercise modalities, including descending stair walking and assisted bodyweight resistance exercise in which concentric loading is reduced, have been shown to improve insulin sensitivity, lipid profiles, and functional strength while imposing minimal cardiovascular load.^104^^,^^105^ The relatively low metabolic demand of these approaches may be advantageous in submarine environments, where hygiene constraints and thermal regulation challenges can make high-sweat activities impractical.^106^ More broadly, eccentric-focused exercise strategies, including eccentric-dominant and eccentric-biased approaches, may offer practical benefits in confined operational settings, as they can be performed quietly and with minimal disruption to surrounding crew and onboard operations, potentially improving acceptability during deployment.

### 4.3 Minimal-exercise dose required for maintenance

Evidence from resistance training and detraining studies indicates that the exercise dose required to preserve muscle mass, strength, and functional capacity following a period of training is substantially lower than that required to induce further, ongoing adaptations.^107^ In younger adults, as little as 1 resistance training session per week, and in some cases a single set performed near failure (ie, when another repetition cannot be completed), has been shown to maintain muscle size and strength for several months following a structured training phase.^108^ In older adults, a higher maintenance volume appears necessary, with studies demonstrating preservation of muscle cross-sectional area using 1 weekly session comprising multiple sets at moderate to high intensities.^81^^,^^109^ Although these findings are derived from land-based resistance training paradigms, they support the broader principle that relatively small, regular mechanical stimuli may be sufficient to preserve musculoskeletal function during periods of constrained activity. This principle is particularly relevant in submarine environments, where exercise opportunities are limited and preventing physical deconditioning is the primary concern.

## 5. Discussion

The findings of this review highlight a consistent pattern across the available literature: submarine deployments are associated with marked reductions in physical activity and a range of physiological changes that, while individually modest in the short term, may collectively increase health risk across a submariner’s career. The evidence supports a trajectory in which repeated exposure to the constrained submarine environment contributes to progressive and potentially cumulative changes in body composition, cardiometabolic health, and musculoskeletal function.

What distinguishes submarine service from other sedentary occupational settings is the concurrent exposure to a cluster of environmental stressors, including elevated CO_2_, vitamin D deficiency from prolonged sunlight absence, disrupted sleep from noncircadian shift schedules, and psychological stress from prolonged isolation, that may independently and synergistically compromise physiological health beyond what physical inactivity alone would predict.^3^ This combination of reduced mechanical loading and adverse environmental conditions creates a uniquely challenging occupational context in which the capacity for physical and physiological recovery is structurally limited, even between deployments.

Several limitations of the existing evidence base deserve acknowledgement. The majority of studies are observational in design, with relatively small sample sizes and considerable heterogeneity in outcome measures, submarine types, and deployment durations. No randomized controlled trials examining exercise interventions in submarine populations were identified; thus, the efficacy of the strategies proposed in this review remains to be established empirically in this context. Most studies have been conducted in male samples from a small number of navies, and generalizability across different submarine fleets, operational cultures, and personnel demographics, including the increasing integration of women into submarine service in several nations, is uncertain. Additionally, the interplay between physical inactivity and other environmental stressors unique to the submarine environment, including sleep disruption, psychological stress, and dietary constraints, has not been comprehensively examined.

Notwithstanding these limitations, the available evidence is sufficient to support the conclusion that maintaining physical activity during submarine deployment is an occupational health priority. The strategies outlined in this review represent evidence-informed approaches that are practically compatible with the submarine environment, and future research should prioritize the development and evaluation of context-specific exercise interventions in this setting. Objective measurement of physical activity during deployment, longitudinal tracking of health outcomes across careers, and investigation of the minimum effective exercise dose for health maintenance in constrained environments represent particularly important research priorities. From an occupational health perspective, these findings reinforce the need for proactive, deployment-specific physical activity strategies that are supported at the command level and integrated into pre- and post-deployment health management.

## 6. Conclusions

Although regular physical activity is fundamental to maintaining health and occupational readiness, exercising during submarine deployments presents unique and substantial challenges. Restricted space, limited equipment availability, demanding shift schedules, and operational requirements to minimize noise and vibration markedly constrain opportunities for structured exercise. Consistent with these constraints, submariners experience pronounced reductions in overall physical activity during deployment, accompanied by high levels of sedentary behavior and reduced exercise participation compared with land-based conditions.

Collectively, the available evidence suggests that prolonged exposure to these conditions may contribute to unfavorable changes in body composition, including increases in fat mass, potential reductions in muscle mass, and in some contexts, alterations in bone health. Importantly, these changes may occur alongside broader physiological adaptations linked to cardiometabolic health, even when short-term clinical markers remain within reference ranges. While the magnitude and persistence of these changes vary across studies, their cumulative effects across repeated deployments may increase long-term cardiometabolic and musculoskeletal risks.

To address these risks, it is essential to identify exercise strategies that are both practical and effective within the submarine setting. Feasible approaches include promoting daily movement to achieve approximately 7500-10 000 steps where operationally possible, integrating no-load resistance exercises (including eccentric-focused approaches), and adopting minimal-dose training protocols that require little time, space, or equipment. Emerging evidence indicates that low-volume exercise strategies can elicit meaningful improvements in strength and physical function with high tolerability and adherence, highlighting their potential as practical countermeasures in highly constrained operational environments. Among these, eccentric-focused approaches may be especially well suited to submarine deployments, as they allow meaningful mechanical loading with relatively low perceived exertion and fatigue. Education around the health benefits of maintaining activity during deployment, alongside visible support from commanding officers, may further facilitate uptake and adherence to these strategies.

## Data Availability

No datasets were generated or analyzed during the current study.
